# Endometriosis increases the risk of gestational diabetes: a meta-analysis stratified by mode of conception, disease localization and severity

**DOI:** 10.1038/s41598-023-35236-y

**Published:** 2023-05-19

**Authors:** Noemi Salmeri, Letizia Li Piani, Paolo Ivo Cavoretto, Edgardo Somigliana, Paola Viganò, Massimo Candiani

**Affiliations:** 1 Gynecology and Obstetrics Unit, IRCCS San Raffaele Scientific Institute, 20132 Milan, Italy; 2 Department of Clinical Sciences and Community Health, Università degli Studi di Milano, 20122 Milan, Italy; 3 Infertility Unit, Fondazione IRCCS Ca’ Granda Ospedale Maggiore Policlinico, Via M. Fanti 6, 20122 Milan, Italy

**Keywords:** Endocrine system and metabolic diseases, Endocrine reproductive disorders

## Abstract

To review the current evidence on the risk of gestational diabetes mellitus (GDM) in women with endometriosis, taking into account relevant confounders such as the higher frequency of Assisted Reproductive Technologies (ART) conceptions. Database searches on PubMed, Medline, Embase and Scopus through June 2022, using combinations of relevant keywords. A total of 18 studies, involving N = 4,600,885 women, were included. The overall risk of GDM in endometriosis patients was significantly higher than in controls (OR, 1.23; 95% CI 1.07–1.51). This significant association persisted in natural pregnancies (OR, 1.08; 95% CI 1.04–1.12) but not in pregnancies conceived through ART (OR, 0.93;95% CI 0.70–1.24). Based on the limited number of studies that examined this association in relation to endometriosis phenotype, an increased risk was found in more severe stages (OR, 3.20; 95% CI 1.20–8.54) but independently from localization of the lesions. Endometriosis increases the risk of GDM, with a possible progressive effect in more advanced stages of the disease. Although the effect magnitude may be limited in some subgroups, this finding has a clinically relevant impact due to both the strong biological plausibility and to the relatively high incidence of both endometriosis and GDM.

## Introduction

Gestational diabetes mellitus (GDM) is one of the most common metabolic disorders in obstetrics and a growing public health concern, given its strong prediction of future type 2 diabetes mellitus (T2DM) in both mothers and infants^1^. The scientific literature defines GDM as a state of hyperglycemia developing in pregnancy and resolving following delivery, caused by insulin resistance or reduced insulin production. Risk factors for GDM encompass pre-pregnancy overweight and obesity, advanced maternal age, excessive weight gain during pregnancy, a family history of T2DM, previous pregnancies with GDM, having given birth to a baby weighing over 4000 g and having multiple pregnancies.

In GDM, the pregnant woman’s metabolism influences both maternal and fetal heath. Excessive carbohydrates/lipids intake raise glucose levels stimulating maternal pancreas to release additional insulin with production of excessive body fat. Immune and inflammatory responses are generated within white adipose tissue, resulting in a low-grade, systemic, chronic metabolic inflammation^2^. The inflammatory response reduces both insulin action and pancreatic β-cell compensatory reaction, promoting the development of GDM^3^. Uncontrolled maternal hyperglycemia contributes to obstetric complications, such as polyhydramnios, macrosomia, labor anomalies or premature birth, to adverse neonatal outcomes, such as hypoglycemia and delayed lung maturation, with long-term consequences for the offspring, like an increased risk of obesity, T2DM and cardiovascular diseases later in life^4^.

GDM is the most frequently reported pregnancy complication among women with polycystic ovary syndrome (PCOS)^5–7^, leading to a three-fold increase in risk^8–11^. PCOS determines pre-existing insulin resistance and compensatory hyperinsulinemia, which lead to hyperandrogenemia that exacerbates hyperglycemia^12–14^. The resulting inflammation impairs β-cell function, further contributing to hyperglycemia during pregnancy^15^.

The rationale for our research question is based upon two observations: (1) the growing interest in adverse pregnancy and neonatal outcomes among women with endometriosis^16–18^; within this line of research, the association between endometriosis and GDM remains unclear. Based on evidence that chronic inflammation and prolonged cytokine exposure increase the risk of GDM^19^, it is plausible that the subclinical inflammation underneath endometriosis might also exhibit this positive association. Notably, available findings are largely influenced by relevant confounders, such as the frequent need of affected women to undergo Assisted Reproductive Technologies (ART) procedures, which ‘per se’ increase the risk of GDM^20^; (2) a novel hypothesis by evolutionary biologists supporting the idea that endometriosis and PCOS represent extreme and diametric (opposite) outcomes of variations in the hypothalamic-pituitary–gonadal axis development and activity, with endometriosis mediated by low prenatal testosterone and PCOS mediated by high prenatal testosterone^21,22^. This diametric disorder hypothesis predicts that women with PCOS and those with endometriosis might display opposite hormonal and metabolic phenotypes also during pregnancy.

The purpose of this systematic review and meta-analysis was to synthesize the best available evidence regarding the association between GDM and endometriosis. The influence of medically assisted reproduction in assessing this association was also considered.

## Results

**Figure 1:**
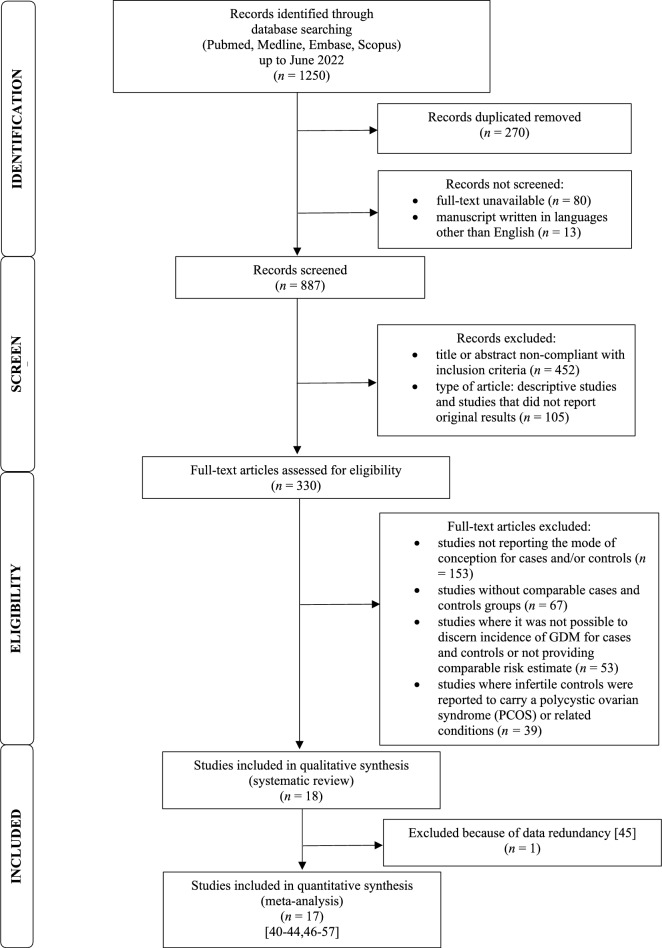
Preferred Reporting Items for Systematic Reviews and Meta-Analyses (PRISMA) flow diagram for study selection.

The PRISMA flow diagram of the review process is illustrated in Fig. 1. Out of the 330 full-text articles evaluated, 312 studies were excluded. In total, 18 studies^23–40^, involving *N* = 4,600,885 women, met the original inclusion criteria. Fifteen cohort studies^23–29,31,32,34–36,38–40^ (*N* = 4,600,016) and 3 case–control studies^30,33,37^ (*N* = 869) were included. Among the cohort studies, 10 were retrospective^23–27,31,34,35,38,40^ (with 2 of them^27,31^ employing a multicentric design), 2 were prospective^32,36^, 2 were based on a historical cohort^28,29^ and 1 was a nationwide study^39^. Out of the 3 case–control studies, 2 had a retrospective design^30,33^, while 1 was a prospective study^37^. A comprehensive summary of the characteristics of the included studies can be found in Table 1.

**Table 1:** Main characteristics of included studies (*n* = 18).

Author, year	Study country	Study design	Study population ^a^	Endometriosis: (1) Diagnosis (2) Localization (3) Stage	Controls ^b^	Mode of conception^c^	GDM diagnosis or definition	Population general characteristics: (1) Age (2) Parity (3) BMI
Kuivasaari-Pirinen et al.^23^	Finland (Europe)	RC	Total: 92; Endo: 49; Controls: 4	(1) LPS or US (2) / (3) /	Male factor infertility	A	Single abnormal value in OGTT	(1) Age: Cases (age > 35 yo): 24.5%; Controls (age > 35 yo): 18.6% (2) Parity: Cases (nulliparous): 83.7%; Controls (nulliparous): 72.1% (3) BMI: Cases (BMI > 25 kg/m^2^): 20.4%; Controls (BMI > 25 kg/m^2^): 22.0%
Mekaru et al.^24^	Japan (Asia)	RC	Total: 88; Endo: 40; Controls: 48	(1) LPS (2) OMA: 8; unknown: 32 (3) stage I: 36; stage II: 5; stage III: 6; stage IV: 2	Women without endo	S	Medical record review	(1) Age: Cases: 33.0 ± 3.8; Controls: 33.6 ± 4.1 (2) Parity: Cases (nulliparous): 55.1%; Controls (nulliparous): 47.5% (3) BMI: /
Aris et al.^25^	Canada (North America)	RC	Total: 31,068; Endo: 784; Controls: 30,284	(1) LPS (2) / (3) /	Women without endo	S + A	1 h OGTT ≥ 10.3 mmol/L (Canadian Guidelines, 2008)	(1) Age: Cases (non complicated/complicated pregnancies): 27.9 ± 5.2/28.5 ± 5.6;—controls (non complicated/complicated pregnancies): 27.1 ± 4.8/27.6 ± 5.4 (2) Parity: / (3) BMI: Cases: 35.77 ± 2.80; Controls: 36.07 ± 3.51
Baggio et al.^26^	Italy (Europe)	RC	Total: 123; Endo: 30; Controls: 93	(1) LPS (segmental bowel resection) (2) all DE (3) /	Healthy women without endo	S + A	Medical record review and database search	(1) Age: Cases: 30.9 ± 3.3; Controls: 30.7 ± 4.0 (2) Parity: / (3) BMI: /
Conti et al.^27^	Italy (Europe)	RC	Total: 2239; Endo: 316; Controls: 1923	(1) Surgery (2) OMA: 35%, OMA + SPE: 25%; OMA + DE: 21%; DE: 19% (3) /	Women without endo	S + A	Carbohydrate intolerance with onset or recognition in pregnancy with positive OGTT	(1) Age^d^: / (2) Parity: Cases (nulliparous): 69.3%; Controls (nulliparous): 69.2% (3) BMI^d^: /
Luke et al.^28^^e^	USA (North America)	HC	Total: 1706; Endo: 295; Controls: 1411	(1) one or more hospital encounters (admissions, observational stays or emergency room visits) (2) / (3) /	Male factor infertility	A	Database search	(1) Age: Cases: 35.1 ± 3.6; Controls: 34.4 ± 4.1 (2) Parity: / (3) BMI: /
Stern et al.^29^^e^	USA (North America)	HC	Total: 300,614 (S: 298,577; A: 2307); Endo: 996 (S: 590; A: 406); Controls: 299,888 (S: 297,987; A: 1901)	(1) one or more hospital encounters (admissions, observational stays or emergency room visits) (2) / (3) /	Male factor infertility (A); fertile women without endo (S)	S + A	Database search or hospital discharge delivery records	(1) Age: Cases (A/S): 35.2 ± 3.6/30.2 ± 5.7; Controls (A/S): 34.4 ± 4.1/29.7 ± 5.8 (2) Parity: / (3) BMI: /
Benaglia et al.^30^	Italy (Europe)	RCC	Total: 478; Endo: 239; Controls: 239	(1) LPS or US for OMA (2) / (3) /	Infertile women without current or past evidence of endo^f^	A	Medical record review and questionnaires for missing data	(1) Age: Cases: 35.5 ± 3.5; Controls: 35.5 ± 3.5 (2) Parity: Cases (no previous deliveries): 90%; Controls (no previous deliveries): 84% (3) BMI: Cases: 21.6 ± 3.1; Controls: 22.5 ± 3.9
Exacoustos et al.^31^	Italy (Europe)	RC	Total: 401; Endo: 101; Controls: 300	(1) Surgery (2) DE nodule ≥ 2 cm remaining after a previously incomplete surgery (3) /	Women without endo	S + A (cases); S (controls)	Carbohydrate intolerance with onset in pregnancy with a positive OGTT	(1) Age^g^: / (2) Parity^g^: / (3) BMI^g^: /
Harada et al.^32^	Japan (Asia)	PC	Total: 9186; Endo: 330; Controls: 8856	(1) Self-administered questionnaire (2) / (3) /	Negative history for endo (self-reported)	S + A^h^	Positive 75 g OGTT (FPG ≥ 92 mg/dL, 1 h PG ≥ 180 mg/dL, and 2 h PG ≥ 153 mg/dL (JSOG and JAOG guidelines)	(1) Age: Cases (yo): < 20: 0.3%; 20–24: 2.7%; 25–29: 15.8%; 30–34: 33.3%; 35–39: 28.4%; ≥ 40: 4.5%; Controls (yo): < 20: 0.9%; 20–24: 7.7%; 25–29: 23.9%; 30–34: 30.8%; 35–39: 18.8%; ≥ 40: 3.4% (2) Parity: Cases (nulliparous): 42.7%; Controls (nulliparous): 36% (3) BMI: /
Jacques et al.^33^	French (Europe)	RCC	Total: 226; Endo: 113; Controls: 113	(1) Surgery or clinical examination and MRI scan (2) OMA: 59.7%; DE: 43.4%; SPE: 41.1% (3) stage I: 20.9%; stage II: 27.9%; stage III: 20.2%; stage IV: 27.1%	Male factor infertility	A	Self-administered questionnaire	(1) Age: Cases: 32.4 ± 3.7; Controls: 31.4 ± 4.2 (2) Parity: Cases (no previous pregnancies): 56.6%; Controls (no previous pregnancies): 57.5% (1) BMI: Cases: 22.0 ± 3.1; Controls: 22.9 ± 3.4
Li et al.^34^	China (Asia)	RC	Total: 375; Endo: 75; Controls: 300	(1) LPS (2) / (3) stage I–II: 54; stage III–IV: 44	Women with no gynecological diseases	S + A	Medical record review	(1) Age: Cases: 32.8 ± 3.4; Controls: 30.1 ± 2.9 (2) Parity: Cases (nulliparous): 78.7%; Controls (nulliparous): 58% (3) BMI: Cases: 21.2 (19.6, 23.0); Controls: 21.5 (19.5, 23.4)
Mannini et al.^35^	Italy (Europe)	RC	Total: 786; Endo: 262; Controls: 524	(1) LPS (2) DE: 15.3%; OMA and/or SPE: 84.7% (3) /	Women without endo^k^	S + A	Positive 75 g 2 h OGTT in a formerly non-diabetic woman after 16 weeks of pregnancy	(1) Age: Cases: 36.89 ± 0.27; Controls: 36.88 ± 0.19 (2) Parity: Cases (nulliparous): 69.1%; Controls (nulliparous): 54% (3) BMI: Cases: 22.18 ± 0.21; Controls: 22.38 ± 0.16
Farland et al.^36^	USA (North America)	PC	Total: 137,635; Endo: 5,665; Controls: 131,970	(1) LPS (2) / (3) /	Non LPS confirmed endometriosis	S	Self-administered questionnaire	(1) Age^i^: Cases: 29.1 ± 5.3; Controls: 29.1 ± 5.3 (2) Parity^i^: Cases (primiparous): 13.8%; Controls (primiparous): 17.9% (3) BMI^i^: Cases: 23.8 ± 4.6; Controls: 23.7 ± 4.6
Sharma et al.^37^	India (Asia)	PCC	Total: 165; Endo: 64; Controls: 101	(1) LPS (2) / (3) all stage III–IV	Tubal factor infertility	A	/	(1) Age: Cases (< 35 yo / ≥ 35 yo): 30.95 ± 2.98/37.25 ± 2.03; Controls (< 35 yo / ≥ 35 yo): 30.64 ± 2.5/36.96 ± 1.8 (2) Parity: / (3) BMI: Cases (< 35 yo/ ≥ 35 yo): 23.6 ± 3.05; 23.56 ± 2.72; Controls (< 35 yo / ≥ 35 yo): 24.39 ± 3.69/24.09 ± 3.42
Warzecha et al.^38^	Poland (Europe)	RC	Total: 360; Endo: 64; Controls: 296	(1) Surgery (2) / (3) stage I: 12.1%; stage II: 30.3%; stage III: 42.4%; stage IV: 15.2%	Healthy fertile women without endo	S + A ^j^	Positive OGTT (PSGO and RCOG guidelines)	(1) Age: Cases: 33.6 ± 4.2; Controls: 31.8 ± 4.6 (2) Parity: Cases (primiparous): 87.5%; Controls (primiparous): 43.9% (3) BMI: Cases: 22.4 ± 3.8; Controls: 23.4 ± 4.6
Epelboin et al.^39^	FRENCH (Europe)	NC	Total: 4,114,833; Endo: 31,101; Controls: 4,083,732	(1) Database search (reported in previous hospitalizations, since 2008) (2) / (3) /	Women without endo	S + A^k^	Database search	(1) Age: Cases (S/A): 31.7 ± 4.8/33.1 ± 4.0; Controls (S): 30.0 ± 5.3 (2) Parity: Cases (primiparous, S/A): 47.39%/76.71%; Controls (primiparous, S): 39.58% (3) BMI: Cases (obesity, S/A): 3.87%/3.10%; Controls (obesity, S): 4.90%
Wang et al.^40^	China (Asia)	RC	Total: 510; Endo: 107; Controls: 403	(1) Database search for infertility diagnosis (2) / (3) /	Infertile male factor	A	Positive OGTT (ACOG guidelines)	(1) Age: Cases: 34.11 ± 3.58; Controls: 33.41 ± 3.50 (2) Parity: Cases (primiparous): 97.2%; Controls (primiparous): 96.5% (3) BMI: Cases (obesity): 0.9%; Controls (obesity): 1.7%

Data are reported as n, %, mean ± standard deviation.

*Endo* endometriosis, *BMI* body mass index, *GDM* gestational diabetes mellitus, *RC* retrospective cohort, *S* spontaneous, *LPS* laparoscopy, *US*, ultrasound, *A* medically assisted, *yo* years old, *OGTT* oral glucose tolerance test, *OMA* ovarian endometrioma, *DE* deep endometriosis, *SPE* superficial peritoneal endometriosis, *HC* historical cohort, *RCC* retrospective case–control, *PC* prospective cohort, *FPG* fasting plasma glucose, *PG* plasma glucose, *JSOG* Japan Society of Obstetrics and Gynecology, *JAOG* Japan Association of Obstetricians and Gynecologists, *MRI* magnetic resonance imaging, *PCC* prospective case–control, *PSGO* Polish Society of Gynecologists and Obstetricians, *RCOG* Royal College of Obstetricians and Gynecologists, *NC* nationwide cohort, *ACOG* American College of Obstetricians and Gynecologists.

^a^Study population were abstracted from original studies according with pre-defined criteria for cases and controls of this meta-analysis.

^b^When original studies reported data stratified by indication to in-vitro fertilization in controls, male factor for infertility was chosen as reference group.

^c^Second-level infertility treatment was considered an exclusion criteria.

^d^Authors declared no statistically significant differences in cases versus controls neither in age nor in BMI.

^e^As redundancy between data from Luke et al.^28^ and Stern et al.^29^ was highly suggested, pooled analysis were performed by omitting the study by Luke et al.^28^.

^f^Data on controls according to the indication for in-vitro fertilization were provided as a single group of control.

^g^Authors declared that cases and controls carried statistically significant differences in terms of age, BMI and parity.

^h^Data on GDM prevalence according with the mode of conception were not provided.

^i^Authors declared that relation between endometriosis and GDM was stronger in pregnancies of women < 35 years, pluriparous and without a history of infertility.

^j^Included also first-level infertility treatments.

^k^Authors excluded pregnancies by assisted reproduction in controls because the cause of infertility was not available.

### Risk of GDM in endometriosis patients versus controls

#### Studies overview

Out of the 18^23–40^ studies included in the qualitative synthesis, two studies^28,29^ were based on the same historical cohort and study period, suggesting a high likelihood of data redundancy. Consequently, the quantitative synthesis was performed by omitting the study by Luke et al.^28^.

The population size of the included studies was highly variable: a total of *N* = 4,114,833 patients (*n* = 31,101 women with endometriosis and *n* = 4,083,732 controls) came from the largest study^39^ whereas only *N* = 88 women (*n* = 40 with endometriosis and *n* = 48 controls) were included in the smallest^24^.

In most of the included studies^23–27,30,31,33–38^, the diagnosis of endometriosis was based on surgical and histological confirmation of the disease. However, only two studies^27,33,35^ provided a complete description of the anatomical localizations of endometriosis lesions, whereas five studies^24,33,34,37,38^ reported data on endometriosis severity according to the revised American Fertility Society (r-AFS) staging system^41^. Notably, two studies focused exclusively on women with deep endometriosis (DE)^26,31^: one study^26^ included women with nodules surgically treated by segmental bowel resection, while another study^31^ evaluated women still exhibiting a posterior DE lesion of at least 2 cm on ultrasound assessment after a previous incomplete surgical excision.

In most studies, controls were defined as women without endometriosis; however, only one study^36^ performed a diagnostic laparoscopy to rule out the disease in controls. Regarding diagnosis of gestational diabetes, 8 studies^23,25,27,31,32,35,38,40^ used a positive oral glucose tolerance test (OGTT) as the criterion. Specifically, one study^25^ followed the Canadian Guidelines^42^, one^32^ followed the Japanese Guidelines^43^, one^38^ adhered to the Polish Guidelines^44^, and another^40^ followed the American Guidelines^45^. Interestingly, one study diagnosed gestational diabetes in cases and controls through self-administered questionnaires^36^; the Authors reported that, according to their previous large prospective cohort study, self-reported adverse pregnancy outcomes are validly reported, with a 94% confirmation rate for GDM^46,47^.

#### Quality assessment

The risk of bias assessment revealed that 8 studies^23,25,32,35–38,40^ were at low risk of bias, 8^26–31,33,34,39^ had a moderate risk of bias, and the remaining one^24^ was at high risk of bias (Supplemental Fig. S1). According to the GRADE approach^48^, the overall quality of the evidence ranged from low to moderate.

#### Data analysis

**Figure 2:**
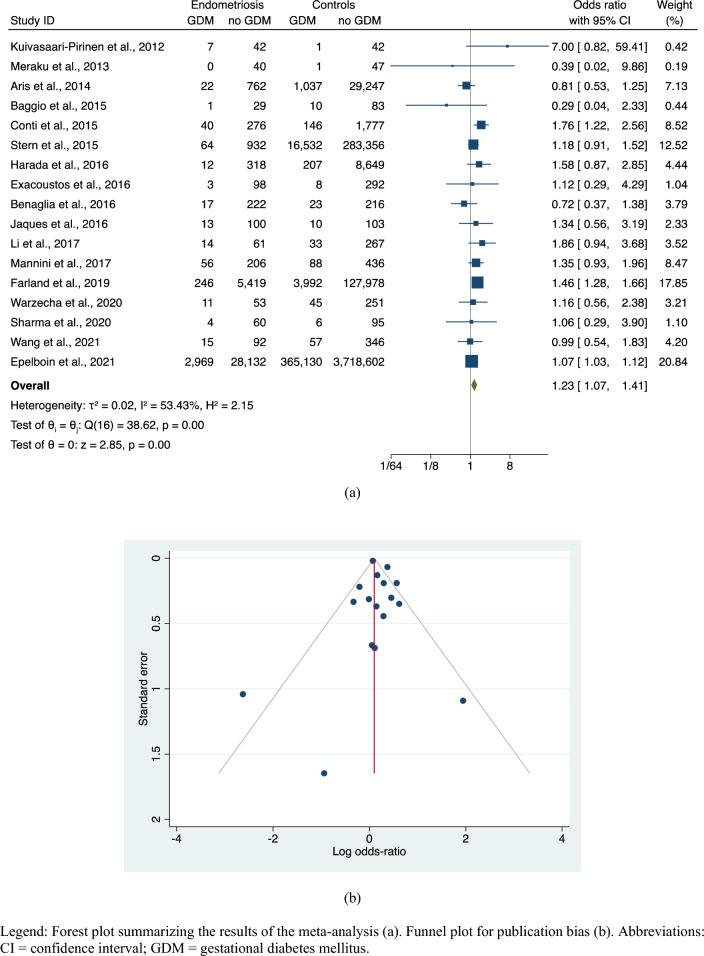
Risk of GDM in endometriosis versus controls. Legend: Forest plot summarizing the results of the meta-analysis (**a**). Funnel plot for publication bias (**b**). Abbreviations: CI = confidence interval; GDM = gestational diabetes mellitus.

The meta-analysis comparing endometriosis cases to controls, regardless the mode of conception, revealed a significantly increased risk of GDM in endometriosis (OR, 1.23; 95% CI 1.07–1.51; 17 studies; *N* = 4,599,449) with moderate heterogeneity (I^2^ = 53.43%) and non-significant publication bias (Egger’s: z = -0.75, *p* = 0.4557; Begg’s: z = -0.87, *p* = 0.4338) (Fig. 2a,b). Sensitivity analyses by omitting one study at time confirmed the robustness of the pooled risk estimate (Supplemental Fig. S2).

### Risk of GDM in endometriosis patients versus controls including only ART pregnancies

In total, 7 studies^23,28–30,33,37,40^ met the inclusion criteria: 2 were retrospective cohort studies^23,40^, 2 were retrospective case–control studies^30,33^, 1 was a prospective case–control study^37^ and 2 were historical cohort studies^28,29^. Population sizes varied significantly: the total sample ranged from *N* = 92 women (*n* = 49 with endometriosis and *n* = 43 controls) in the smallest study^23^ to *N* = 2,307 women (*n* = 406 with endometriosis and *n* = 1,901 controls) in the largest^29^.

Most included studies^23,29,30,33^ considered ART cycles with pregnancies achieved through both fresh and frozen embryo transfers. One study included only fresh cycles^37^, and another included only frozen ART cycles^40^. For the majority of the included studies, we were able to extract data on our primary outcome specifically for male factor infertility controls^23,29,33,40^. General characteristics of the included studies are summarized in Table 1.

#### Quality assessment

Three studies^23,37,40^ were judged at low risk of bias, and three^29,30,33^ had a moderate risk of bias (Supplemental Fig. S1). The overall quality of the evidence, according to the GRADE approach^48^, was deemed low.

#### Data analysis

**Figure 3:**
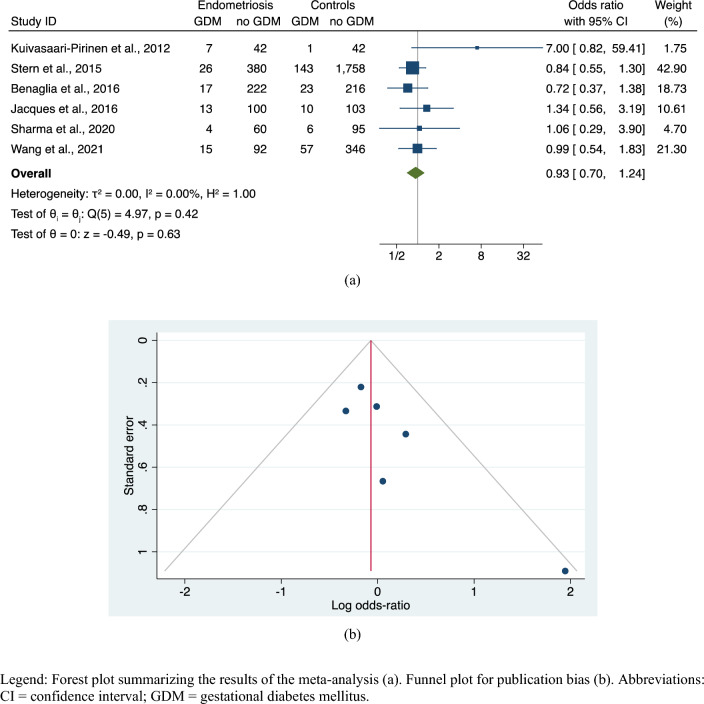
Risk of GDM in endometriosis versus controls, only pregnancies by ART. Legend: Forest plot summarizing the results of the meta-analysis (**a**). Funnel plot for publication bias (**b**). Abbreviations: CI = confidence interval; GDM = gestational diabetes mellitus.

The meta-analysis failed to reveal significant differences in GDM risk between women with endometriosis and controls in the ART population (OR, 0.93; 95% CI 0.70–1.24; 6 studies; *N* = 3,778; *p* = 0.63) (Fig. 3a), with no heterogeneity (I^2^ = 0%). Symmetry was observed upon visual inspection of the funnel plot (Fig. 3b), and both Egger’s test (z = 1.76, *p* = 0.0787) and Begg’s test (z = 1.13, *p* = 0.2597) showed no evidence of a small-study effect. Subgroup analyses according to predefined moderators did not find any group difference in the pooled risk estimates (Supplemental Fig. S3).

### Risk of GDM in endometriosis patients versus controls including only spontaneous pregnancies

#### Studies overview

Overall, only 3 studies^24,29,39^ could be included in this comparison: one was a retrospective cohort study^24^, one was a historical cohort^29^ and one was a nationwide cohort study^39^. The total population size was however quite large (*N* = 4,413,498).

Notably, the study population of Mekaru et al.^24^ also included conceptions through first-level infertility treatments (ovulation induction and intrauterine insemination). However, infertility treatments were reported to be comparable between the groups. Since conceptions through in-vitro fertilization (IVF) were excluded a priori, we did not consider any of the pregnancies evaluated by Mekaru et al.^24^ as obtained by ART. General characteristics of the studies included in this comparison are summarized in Table 1.

#### Quality assessment

Two studies were at moderate risk of bias^29,39^ and one had a high risk of bias^24^ (Supplemental Fig. S1). The overall quality of the evidence, according to the GRADE approach^48^, was deemed low.

#### Data analysis

**Figure 4:**
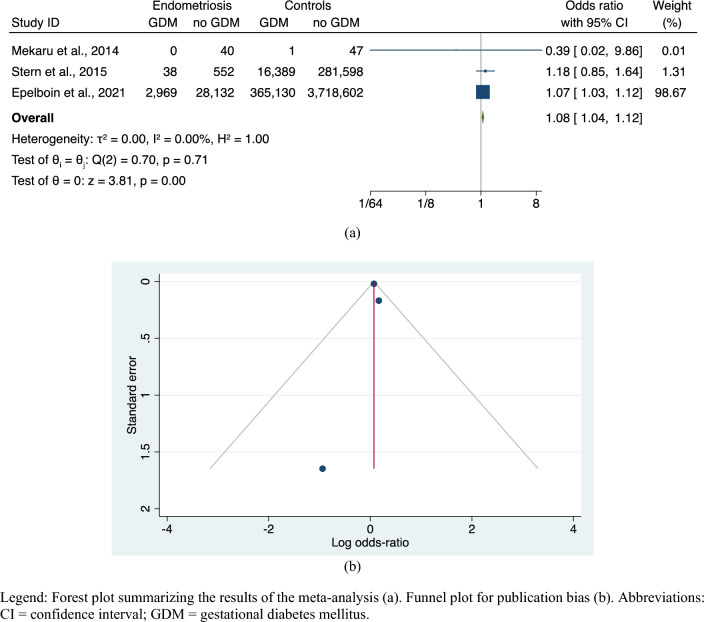
Risk of GDM in endometriosis versus controls, only spontaneous pregnancies. Legend: Forest plot summarizing the results of the meta-analysis (**a**). Funnel plot for publication bias (**b**). Abbreviations: CI = confidence interval; GDM = gestational diabetes mellitus.

The meta-analysis revealed a significantly increased risk of GDM in endometriosis women compared to controls in the natural conception population (OR, 1.08; 95% CI 1.04–1.12; 3 studies; N = 4,413,498; *p* < 0.001) (Fig. 4a), with no heterogeneity (I^2^ = 0%) and a non-significant small-studies effect (Egger’s: z = − 0.08, *p* = 0.934; Begg’s: z = − 1.04, *p* = 1.00) (Fig. 4b).

### Risk of GDM in endometriosis patients comparing ART and natural pregnancies

#### Studies overview

Overall, 4 studies met the inclusion criteria^29,34,38,39^: one was a historical cohort^29^, two were retrospective cohort studies^34,38^, and the remaining was a nationwide cohort study^39^. Study populations were smaller than those in other comparisons performed, yet sample size varied considerably between studies: the largest study^39^ assessed a total of N = 38,035 patients (n = 6,934 endometriosis women who conceived by ART and n = 31,101 who conceived naturally), while the smallest^38^ included only N = 64 women (n = 36 endometriosis women with ART conceptions and n = 28 endometriosis women with natural conception).

Of all, 2 studies^29,39^ included ART cycles with pregnancies obtained by both fresh and frozen embryo transfers; the remaining two studies^34,38^ did not mention if ART cycles included only fresh or only frozen embryo transfers or both. Notably, data from Warzecha et al.^38^ for natural conception also included first-level infertility treatments (intrauterine insemination). General characteristics of the included studies are summarized in Table 1.

#### Quality assessment

Overall, one study was at low risk of bias^38^, while the remaining three were at moderate risk of bias^29,34,39^ (Supplemental Fig. S1). The overall quality, as judged by the GRADE approach^48^, was considered low or very low.

#### Data analysis

**Figure 5:**
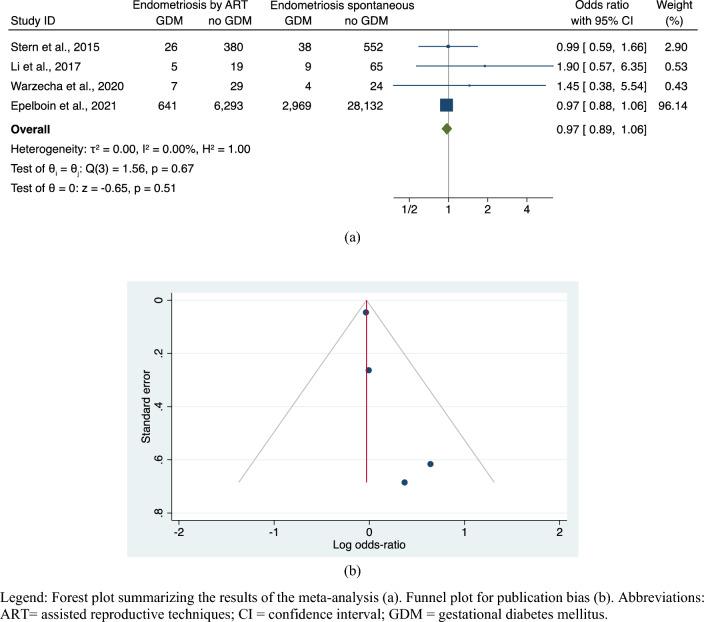
Risk of GDM in endometriosis pregnancies by ART versus endometriosis spontaneous pregnancies. Legend: Forest plot summarizing the results of the meta-analysis (**a**). Funnel plot for publication bias (**b**). Abbreviations: ART = assisted reproductive techniques; CI = confidence interval; GDM = gestational diabetes mellitus.

The meta-analysis failed to find any significant difference in GDM risk in pregnancies of patients affected by endometriosis with different modes of conception (ART versus natural) (OR, 0.97; 95% CI 0.89–1.06; 4 studies; N = 39,193; *p* = 0.51), with no heterogeneity in pooling data (I^2^ = 0%) (Fig. 5a). Symmetry of funnel plot and non-significant tests for small-studies effect (Egger’s: z = 1.06, *p* = 0.287; Begg’s: z = 1.02, *p* = 0.308) showed absence of significant publication biases (Fig. 5b).

### Risk of GDM in patients with deep endometriosis versus all other localization of endometriosis

#### Studies overview

Overall, only 2 studies^33,35^ provided data on the prevalence of GDM in DE compared to all other localizations of endometriosis (ovarian and/or superficial and/or DE with concomitant ovarian and/or superficial lesions). Both had a retrospective design; one was a cohort study^35^ and one was a case–control study^33^. The overall study population was relatively small (*N* = 350). Interestingly, the prevalence of DE in the original study populations was 15.3% in the study by Mannini et al.^35^ and 43.4% in that by Jacques et al.^33^. General characteristics of the included studies are summarized in Table 1.

#### Quality assessment

Of the two included studies, one was at low risk of bias^35^ and the other was at moderate risk of bias^33^ (Supplemental Fig. S1). The overall quality of the evidence, according to the GRADE^48^ approach, was judged as very low.

#### Data analysis

**Figure 6:**
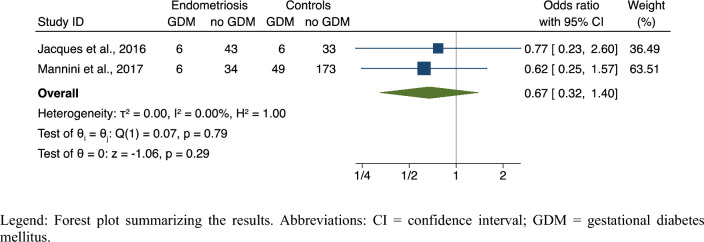
Risk of GDM in deep endometriosis versus all other localization of endometriosis. Legend: Forest plot summarizing the results. Abbreviations: CI = confidence interval; GDM = gestational diabetes mellitus.

The meta-analysis failed to show a significant difference in the risk of GDM in pregnant patients with different localizations of endometriosis: DE versus other disease localizations (OR, 0.67; 95% CI 0.32–1.40; 2 studies; N = 350; *p* = 0.29), with no heterogeneity (I^2^ = 0%) (Fig. 6). Relative symmetry was observed on visual inspection of the funnel plot (data not shown); however, due to the very low number of publications, publication bias could not be entirely ruled out.

### Risk of GDM in stage III-IV versus stage I-II endometriosis

#### Studies overview

Overall, only two studies provided complete data on the prevalence of GDM according to endometriosis severity^33,38^. One was a retrospective cohort study^38^ and the other was a retrospective case–control study^33^. The total study population was very small (*N* = 175). The prevalence of stage III–IV endometriosis patients according to the r-AFS classification^41^ was similar in the two study populations: 57.81% in Warzecha et al.’s cohort^38^ and 46.85% in Jacques et al.’s sample^33^. General characteristics of the two included studies are summarized in Table 1.

#### Quality assessment

One study was at low risk of bias^38^, and the other study was at moderate risk of bias^33^ (Supplemental Fig. S1). Due to the very low number of publications available, the overall quality of the evidence, as judged by the GRADE approach^48^, was considered very low.

#### Data analysis

**Figure 7:**
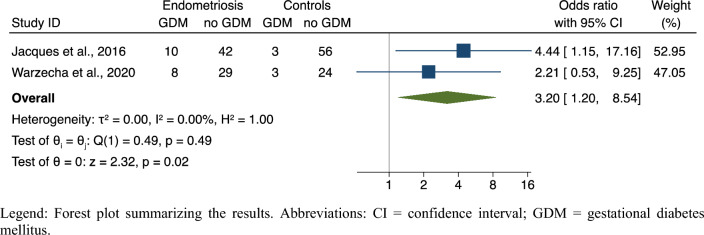
Risk of GDM in stage III–IV versus stage I–II endometriosis. Legend: Forest plot summarizing the results. Abbreviations: CI = confidence interval; GDM = gestational diabetes mellitus.

The meta-analysis found a significantly increased risk of GDM in patients with stage III-IV disease severity compared to stage I-II (OR, 3.20; 95% CI 1.20–8.54; 2 studies; N = 175; *p* = 0.02). Patients with advanced stages of the disease showed more than threefold increase in the risk of the outcome, with no study heterogeneity (I^2^ = 0%) (Fig. 7). Relative symmetry was observed upon visual inspection of the funnel plot (data not shown), demonstrating non-significant evidence of publication bias despite the very low number of publications available.

## Discussion

This meta-analysis demonstrates that the GDM risk is increased in pregnancies with endometriosis compared to unaffected controls. We could observe a noteworthy sequence of progression with significantly greater risk of GDM in more severe stages of endometriosis. The association of endometriosis and GDM remained stable in most subgroups analyses, including those related to study design, method of diagnosis of endometriosis and/or of GDM. Overall, the association of endometriosis and GDM appeared unrelated to the method of conception, given the absence of significance difference in the risk when comparing endometriosis patients conceiving spontaneously with those achieving pregnancy by medically assisted reproduction. The overall quality of the evidence for the main comparison according to GRADE approach^48^ is low to moderate, heterogeneity is low-moderate, and publication bias or small-study effects were not demonstrated.

Endometriosis has been consistently associated with several adverse pregnancy outcomes, and mounting evidence suggests an increased risk especially of preterm birth, pregnancy hypertension and small-for-gestational age^16,17^. This comes in line with our results, given the known association of some of these unfavorable pregnancy outcomes with GDM.

However, to date, the real association between endometriosis and many pregnancy complications remains rather controversial. The fact that the most women with endometriosis suffer from infertility and are thus ART-users is probably the main driver of such discordant findings. Indeed, women conceiving by ART are known to be at high risk for several obstetric complications, including GDM^20^, independently of the cause of infertility.

To account for ART influence on pooled risk estimates, in this meta-analysis we stratified comparisons of endometriosis to unaffected controls according to the method of conception; interestingly, results remained stable only when accounting for spontaneous conceptions. Infertile PCOS women, who are known to carry a higher risk of GDM^7–12^, are often ART users. The possibility of PCOS as indication for ART in our control population is unlikely, as we have excluded controls affected by this disease (unless they were undetectable from other causes of infertility in original studies). On the other hand, ovulatory disorders, in general, have been shown to be associated with GDM^49^ and could have been represented consistently within the control groups of the ART studies. In any case, our results confirm the suggested role of ART as a major confounder in interpreting current research data and the imperative need to weigh comparisons according to this eventual parallel risk factor for GDM in controls.

Interestingly, unlike pregnancy outcomes related to placental dysfunctions in which endometriosis and ART conceptions somehow present additive risks^50^, this does not seem to happen for GDM. Our findings, supporting no differences in risk estimates between endometriosis pregnancies conceived naturally or by ART, strengthen the idea that endometriosis itself and not ART treatment determines an increase in GDM risk in women affected by the disease. This is in line with recent findings suggesting that endometriosis is associated with adverse pregnancy outcomes independently from infertility diagnosis or fertility treatment^51^.

An effect gradient between r-AFS stage^41^ of endometriosis and GDM was observed in this meta-analysis. This is quite interesting considering that endometriosis progression has been related to increased levels of circulating and peritoneal fluid interleukins, systemic inflammation, and immune activation, with an overall higher prevalence of autoimmune diseases^52^.

The possible etiology of GDM in endometriosis patients is likely linked to the systemic inflammation associated with the disease^53,54^. GDM itself is not only related to increased insulin resistance and glucose intolerance, but also to low-grade systemic inflammation^55^. While adipose tissue is increasingly recognized as a legitimate immune organ in PCOS patients, in endometriosis patients, the disease itself contributes to the production of inflammatory effectors such as leptin, tumor necrosis factor-alpha and interleukin-6 with reduced production of adiponectin, potentially leading to insulin resistance. Leptin levels are increased and those of adiponectin decreased in women with endometriosis^56^, particularly during pregnancy when the mother frequently increases carbohydrates intake.

Our results do not seem to support the diametric model proposed by the Crespi’s group^21,22^, which suggests that PCOS and endometriosis would arise as maladaptive extremes due to variation in hypothalamic–pituitary–gonadal axis development and intrauterine androgens levels. According to this hypothesis, the metabolic and endocrine alterations observed in PCOS, such as GDM, would not be present in women with endometriosis^21,22^. On the other hand, we cannot exclude that GDM in endometriosis arises as a phenomenon secondary to sustained inflammation and immune dysregulation and therefore would be totally unrelated to the mechanisms underlying the disease etiology. Indeed, endometriosis is a lifelong disease in which chronic inflammation acts as one of the main drivers possibly involved not only in the genesis and maintenance of endometrial ectopic lesions, but also in the establishment of a susceptibility status for several comorbidities in the life course of women affected.

The major strength of our work is the high biological plausibility justifying the association found, and the fact that the incidence of both endometriosis and GDM is such that the emerged risk increase has a significant impact on clinical practice. The finding that endometriosis increases GDM risk further supports the idea that women with endometriosis may represent a unique population at greater risk for adverse outcomes across pregnancy. If currently GDM diagnosis is based on evaluation of blood glucose levels at late stages of pregnancy, the presence of endometriosis should potentially modify this criterion. Therefore, endometriosis may be considered as a red flag and should be included among the routinely early assessed risk factors for GDM. In this sense, the earlier and more specific detection of GDM in women with endometriosis could improve pregnancy management and final maternal–fetal outcomes. Indeed, elucidating pathways for prevention, screening and intervention in pregnancies of women with endometriosis will be critical to improve the health of these women and their children.

This study presents other important strengths. First, only one meta-analysis^57^ to date has specifically investigated the association between endometriosis and GDM, concluding that endometriosis had no significant effect on GDM risk. Our analysis has several added values, including the fact that we have incorporated more recent data. Even more importantly, the novelty of our meta-analysis is that results were provided weighting and stratifying the estimates accounting for clinically relevant confounders, managing the possible over-estimate of the effect at a population level. Third, the estimation of the certainty of the evidence following GRADE guidelines^58^ allowed us to identify missing gaps in current knowledge. Forth, the estimation method^59^ adopted allowed us to produce a robust, unbiased, nonnegative estimate of between-study variability. In this meta-analysis there are also some limitations that must be acknowledged. First, the evidence was mainly generated by observational studies, so the quality of the evidence is moderate to low according to GRADE guidelines^58^. Secondly, sample sizes of original studies were quite heterogeneous, with very large study populations that could have strongly influenced our results. To limit the larger weight in risk estimate from larger studies, sensitivity analysis was obtained by omitting one study at time, giving consistent results. Thirdly, substantial heterogeneity across studies was observed particularly in the population under study and the definition of endometriosis and/or GDM. These limitations were managed with subgroup analysis, even if the number of studies available was limited and the resulting quality of the evidence was graded as low, hindering generalization of some results. This suggests that further research is needed, possibly standardizing the reporting of disease prevalence by endorsing major international guidelines to reach more robust conclusions.

In conclusion, this systematic review and meta-analysis showed that endometriosis is associated with an increased rate of GDM. Therefore, a positive anamnesis for endometriosis must be considered in the prevention, early diagnosis and management of GDM, both in clinical practice and in research settings. It is important to consider the risk of other coexisting conditions frequently encountered in patients with endometriosis, such as autoimmune diseases, as clearly these risks contribute to the risk of adverse pregnancy outcomes^60^. We can also speculate on the possibility of GDM prevention based upon adequate treatment of endometriosis with pharmacological or surgical methods. More research is required to examine this topic in more detail, including investigations of the underlying mechanisms explaining the association of endometriosis with GDM.

## Methods

This is a systematic review and meta-analysis performed according to the Preferred Reporting Item for Systematic Reviews and Meta-analysis (PRISMA)^61^ and the Meta-analysis Of Observational Studies in Epidemiology (MOOSE)^62^ guidelines. The study protocol was prospectively registered (date registered: March 13, 2022) on the publicly accessible database PROSPERO with the registration ID CRD42022309113.

### Eligibility criteria, information sources, search strategy

A systematic literature search from April 2022 through June 2022 was performed using the following databases: PubMed, Medline, Embase and Scopus. MeSH terms for PubMed and comparable terms for other databases were used. Literature search was based on the following search terms’ combination: ((gestational diabetes) OR (pregnancy diabetes) OR (pregnancy complication) OR (maternal outcomes) OR (pregnancy out-comes)) AND ((endometriosis) OR (endometrioma)) AND ((IVF) OR (ICSI) OR (ART) OR (natural conception) OR (spontaneous conception)) and limited to studies on humans. No restrictions for year of publication and geographic location were applied. Only full-length manuscripts written in English language and published in peer-reviewed journals were screened. Bibliography of relevant papers was also examined to identify any relevant article not captured by the electronic searches. Duplicates were removed by Endnote Software (available online: https://endnote.com, accessed on 02 July 2022). The literature search and the article eligibility were independently assessed by two Authors (N.S. and L.L.P). Disagreements were resolved by discussion with a third reviewer (P.V.).

### Inclusion and exclusion criteria

Case–control and cohort studies reporting the incidence of GDM in pregnant women with diagnosis of endometriosis compared with a control group were included. We did not include descriptive studies (case-reports and case-series) and studies that did not reported original results (reviews, abstracts, editorials, comments).

We included only original studies reporting a confirmed diagnosis of endometriosis in cases; women with endometriosis were included regardless of their medical/surgical treatment history or symptoms before pregnancy. The controls were women without a diagnosis of endometriosis, including both fertile and infertile women referred to a specialized Fertility Centre. When original studies reported data in controls stratified by specific indications for ART, we used the reference group whose infertility was due to male factor as the first option or, as a second option, to other indications for ART with no diagnosis of endometriosis. Male factor infertility was used as a reference group in several other studies of ART outcomes^63–66^, suggesting absence of infertility issues for the female partner (misdiagnosis of endometriosis is expected to be below 5%: similar to the general population). Controls with PCOS diagnosis were always excluded, in light of our research question. Additionally, studies where the entire population of controls was reported to have PCOS and/or altered glucose tolerance or insulin-resistance were a priori excluded. Studies in which for controls it was impossible to ascertain that infertility was unrelated to endometriosis were excluded.

Medically assisted reproduction was defined as a pregnancy achieved by second-line ART treatments, including IVF or intracytoplasmic sperm injections (ICSI) procedures. Studies not mentioning the mode of conception (either natural or medically assisted or both) for endometriosis cases and/or controls were excluded.

### Data extraction

Data from original studies were extracted by two independent reviewers (N.S. and L.L.P). The following data were collected and tabulated: author; publication year; study country; study design; sample size; frequency of GDM in cases and controls; diagnostic modality for endometriosis; criteria followed to define gestational diabetes; endometriosis localization (ovarian, superficial, deep); endometriosis severity according to r-AFS^41^; type of controls (fertile or infertile, infertility etiology); mode of conception (natural or medically assisted); type of ART (IVF/ICSI, fresh or frozen embryo transfer); demographic data (maternal age, body mass index (BMI) and parity); other possible confounding variables at multivariate analysis.

### Assessment of risk of bias

The risk of bias within and across studies was assessed referring to the Risk of Bias In Non-randomized Studies of Exposures (ROBINS-E) tool from the updated Cochrane collaboration guidelines^67^. Based on answers to the signaling questions of the seven bias domains for ROBINS-E tool^68^, an overall judgment was reached so that each study was classified as follows: low risk of bias, when the study was considered comparable to a well-performed randomized trial; moderate risk of bias, for studies providing sound evidence for a non-randomized study but still not comparable to those coming from a well-performed randomized trial; serious risk of bias, when the study had one or more important problems (serious risk of bias in at least one domain, but not at critical risk of bias in any domain); critical risk of bias, when the study was judged as too problematic to provide any useful evidence.

To guide interpretation of the confidence in the effect estimates, the certainty of the evidence was graded into four levels according to the Grading of Recommendations Assessment, Development and Evaluation (GRADE) guidelines^58^: high, moderate, low or very low. Risk of bias was evaluated by two independent reviewers (N.S. and L.L.P); where disagreement occurred, consensus was reached with input from a third team member (P.V.).

### Study outcomes and outcomes measures

The a priori planned primary outcome of the current meta-analysis was the incidence of GDM in pregnancies from endometriosis patients compared to unaffected controls. Original studies enrolling women with pre-gestational diabetes or conditions characterized by altered glucose metabolism not satisfying the diagnosis of GDM (i.e., impaired fasting glucose or impaired glucose tolerance) were excluded. Also, according with American Diabetes Association^69^, studies where the diagnosis of diabetes occurred during the 1st trimester of pregnancy were not included. In light of the absence of a worldwide adopted diagnostic criteria for GDM, we planned a subgroup analysis for the primary outcome according with the diagnostic modality for GDM reported in original studies.

### Meta-analysis methods

#### Data synthesis

To provide a theoretical underpinning of the qualitative synthesis, a quantitative synthesis of included studies was also performed by an independent reviewer (N.S.). Original data on binary outcome measures were extrapolated so that Log Odds Ratios (ORs) with 95% Confidence Intervals (CIs) and corresponding standard errors (SEs) were computed from original data and pooled together. A random-effects (RE) meta-analysis model was performed to estimate pooled effect sizes and a restricted maximum likelihood (REML) estimation method was used to compute between-study variabilities ($\uptau _{2}$)^59^. When the assumption of study homogeneity was reasonable, a fixed-effects (FE) model using Mantel–Haenszel method was also performed^70^.

The overall effect size was estimated as the weighted average of study-specific effect sizes, with larger studies having greater weights and smaller studies having lesser weights. Forest plots were used to present study-specific and overall effect sizes with their respective CIs. Sensitivity analyses were conducted by omitting one study at time to present relative influence of each study on pooled risk estimate.

STATA version 17 software (Stata Corp LLC, 2021, College Station, TX, USA) was used for all statistical analyses. A p-value < 0.05 was considered to be statistically significant.

#### Groups’ comparisons

The primary analysis to answer the research question was obtained comparing pooled data on the risk of GDM in endometriosis patients versus controls, independently from mode of conception (both medically assisted and spontaneous). Pooled risk estimates for the primary outcome were also provided separately according to the following groups comparisons: (1) pregnancies from endometriosis patients conceived by ART versus pregnancies from controls conceived by ART; (2) spontaneous pregnancies in endometriosis women versus spontaneous pregnancies in controls; (3) pregnancies in endometriosis patients conceived by ART versus pregnancies in endometriosis cases conceived by natural conception; (4) DE versus all other localizations of endometriosis; (5) cases affected by stage III-IV endometriosis versus stage I-II endometriosis.

#### Assessment of publication bias and small-study effects

To investigate the impact of publication bias and small-study effects on final results, funnel plots were implemented scattering the logarithm of the study-specific effect sizes (log ORs) against their SEs. Funnel plot asymmetry was tested using both the linear regression-based method according to Egger et al.^71^ and the adjusted rank correlation test proposed by Begg et al.^72^.

#### Assessment of heterogeneity

Between-study heterogeneity was explored throughout the I^2^ statistics which estimates the percentage of total variation across studies that is due to between-studies heterogeneity rather than to sampling variation^73^. I^2^ index values were interpreted as follows: 0–25%, insignificant heterogeneity; 25–50%, low heterogeneity; 50–75%, moderate heterogeneity; > 75%, high heterogeneity^1^. The Chi-squared statistic was also interpreted as a result of heterogeneity, so that a low p-value (< 0.10) questioned the validity of the pooled risk estimates^74^.

#### Subgroup analysis and investigation of heterogeneity

Subgroup-analyses were performed according to Wang et al.^75^ to explore the level of heterogeneity explained by study-level covariates. We planned to carry out subgroup-analyses accounting for the following study moderators: study design, study country, endometriosis diagnosis (self-reported/questionnaire or surgical/histological), GDM diagnosis (medical record review/database search/questionnaire or positive glucose tolerance test or unknown), age of population (≤ 35 years or > 35 years or unknown), BMI categories (normal or unknown) and parity (both multiparous and nulliparous or only nulliparous). For subgroup-analyses performed selectively on ART population, the following covariates were also included: type of control for endometriosis cases (all causes of infertility or only male factor) and type of ART cycle (fresh and frozen or only fresh or only frozen).

## Supplementary Information

Supplementary Information.

## Data Availability

The data for this meta-analysis are freely available. The PROSPERO protocol can be found at https://www.crd.york.ac.uk/prospero CRD ID: CRD42022309113.
