# Supplementary material for: Endometriosis increases the risk of gestational diabetes: a meta-analysis stratified by mode of conception, disease localization and severity
Source: Sci Rep. 2023 May 19;13:8099. doi: 10.1038/s41598-023-35236-y (PMC10199077; doi:10.1038/s41598-023-35236-y)
Supplement: Supplementary file 1 — Supplementary Information. [file 41598_2023_35236_MOESM1_ESM.docx]

**Supplemental Fig. S1** Risk Of Bias In Non-randomized Studies of Exposures (ROBINS-E) tool: traffic light summary chart.


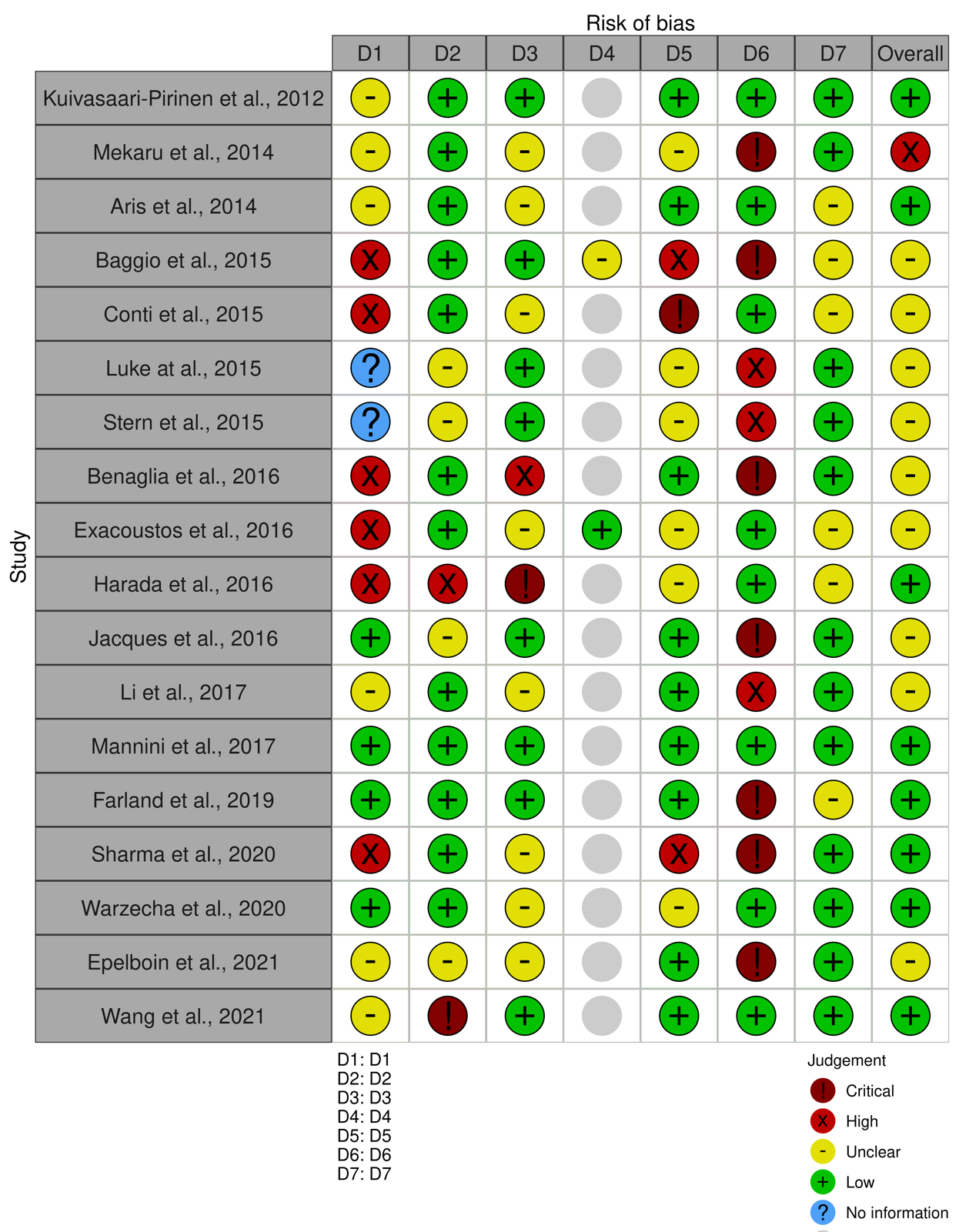


Legend: D1: Bias due to confounding; D2: Bias arising from measurement of the exposure; D3: Bias in selection of participants into the study (or into the analysis); D4: Bias due to post-exposure interventions; D5: Bias due to missing data; D6: Bias arising from measurement of the outcome; D7: Bias in selection of the reported result.

**Supplemental Fig. S2** Sensitivity analysis for primary analysis: leave-one-out meta-analysis.

Abbreviations: CI = confidence interval.

**Supplemental Fig. S3** Subgroup-analyses: endometriosis versus controls in ART population.

Legend: Forest plot summarizing the results of subgroup-analyses. Abbreviations: CI = confidence interval; ART = assisted reproductive techniques; GDM = gestational diabetes mellitus; BMI = body mass index.
